# Spatial ecology meets quality control: a GIS-integrated strategy for visualizing and managing microbial contamination in sterile pharmaceutical cleanrooms

**DOI:** 10.1128/spectrum.00268-26

**Published:** 2026-06-15

**Authors:** Jiaji Wang, Pan Jiang, Junhui Yan, Huili Shen, Lajie Wu, Fen Wei, Xiaozhen Lin, Leiming Xu

**Affiliations:** 1 Beijing Zhifei Lvzhu Biopharmaceutical Co., Ltd., Beijing, China; 2 Anhui Zhifei Longcom Biopharmaceutical Co., Ltd. 733192 , Hefei, China; 3 Anhui Institute for Food and Drug Control, Hefei, China; Connecticut Agricultural Experiment Station, New Haven, Connecticut, USA

**Keywords:** sterile pharmaceutical, clean area, microorganisms, spatial visualization, contamination control, geographic information system

## Abstract

To enhance contamination source identification in sterile drug manufacturing, this study innovatively developed an integrated strategy combining geographic information system (GIS) spatial visualization with microbial contamination control. Between 2022 and 2025, researchers collected 1,117 environmental microbial isolates from sterile preparation workshops, analyzing their population structure, distribution patterns, and potential risks through 16S rRNA/ITS sequencing. GIS technology was employed to associate strain data with workshop spatial information, thereby providing a visual representation of microbial quantity, species composition, and distribution patterns. Results showed that *Staphylococcus* and *Micrococcus* dominated in clean areas, with microbial diversity highest in Controlled Not Classified (CNC) environments and lowest in A-grade areas. The microbial community structure in A-grade areas significantly differed from that in CNC/C/B-grade areas, while CNC/C/B-grade areas exhibited relative similarity. In the case study, the environmental microbial distribution maps clearly demonstrated regional variations and aggregation patterns. By identifying critical control areas and transmission pathways through contamination risk analysis, targeted interventions were designed and implemented, reducing the microbial contamination rate in target C-grade areas from 4.3% to 2.2%, thereby validating the strategy’s effectiveness. This study targets the deficiency of “spatial visualization analysis” in clean area environmental monitoring. The proposed comprehensive strategy effectively fills the methodology gap in spatial analysis and contamination control for current clean area microbial monitoring. It provides a feasible framework for transforming environmental monitoring in the pharmaceutical industry from a passive surveillance system to an active early-warning system, assisting in enhancing the sterility assurance level of pharmaceutical production.

**IMPORTANCE:**

Analyzing the spatial distribution characteristics of microorganisms is crucial for developing effective pollution control strategies. However, existing environmental monitoring methods have limitations in revealing these spatial distribution patterns. This paper proposes an innovative strategy that integrates geographic information system (GIS) spatial analysis with microbial ecology research to enhance the accuracy and scientific rigor of pollution source identification and risk control. This approach enables the visualization of environmental microbial quantities, types, and spatial distribution, providing a quantitative tool for analyzing microbial contamination patterns and tracing transmission pathways. The developed “GIS-integrated strategy” methodology promotes a paradigm shift from merely confirming “microbial presence” to systematically analyzing the multidimensional relationships among “microorganism-environment-control.” This study not only provides a scientific basis for formulating pollution control protocols in the pharmaceutical industry, contributing to improved sterility assurance, but also serves as a practical example of interdisciplinary integration between microbial ecology and spatial information science, demonstrating significant theoretical value and industry application prospects.

## INTRODUCTION

The control of microbial contamination during the production of sterile pharmaceutical products, especially cellular biological products that are highly sensitive to heat or radiation, is a key step to ensure patient safety and product efficacy. Due to the inability to perform terminal sterilization, the entire manufacturing process relies on a stringent environmental control strategy. As a result, environmental monitoring of clean areas for aseptic production is mandatory by national drug regulatory authorities through current Good Manufacturing Practice (1, 2).

Notably, despite technological advancements driving continuous improvement in pharmaceutical quality management systems, deficiencies in sterility assurance and microbial contamination remain the leading cause of drug recalls over the past 5–10 years (3–6). This persistent challenge underscores the critical need to deepen our understanding of microbial behavior within manufacturing environments.

However, effective contamination control requires not only identifying “what microorganisms are present” but also elucidating “where they are located” and “why they persist in specific locations.” This necessitates systematic characterization of microbial spatial distribution patterns, which is critical for evaluating the efficacy of contamination control strategies. Such spatial analysis can reveal microbial cross-contamination events, identify transmission pathways, and thereby support the development of targeted corrective and preventive actions. Furthermore, it provides scientifically defensible data for investigating environmental monitoring excursions and product contamination incidents (7–9).

Currently, research on environmental monitoring largely relies on traditional culture-based methods and molecular biology techniques (such as 16S rRNA sequencing) for microbial identification and frequency counts (10–13). Although these methods provide valuable information on microbial composition, they have inherent limitations in revealing the spatial distribution characteristics of microbes. Most research data are derived from a single cleanliness level or a localized functional area, lacking systematic spatial comparisons across different levels. The results are often presented in tabular form or simple charts, which fail to intuitively display the clustering, gradient, and correlation of microbial contamination in physical space. As a result, the characterization of microbial spatial differences remains at the level of abstract data description. The identification of contamination sources relies heavily on manual experience and speculation, leading to high variability and limited ability to trace contamination transmission pathways—ultimately constraining the scientific rigor of risk assessments.

At its core, a common industry bottleneck stems from the disconnect between microbial data and critical environmental information, such as spatial distribution, personnel flows, and material movements. This disjunction prevents the practical application of monitoring data, rendering monitoring results ineffective in guiding targeted contamination prevention and control measures. Although next-generation sequencing technologies, particularly whole-genome sequencing (WGS), have demonstrated powerful capabilities in precisely tracing contamination events (14, 15), their high costs, complex operations, and lengthy turnaround times make them impractical for routine, large-scale spatial risk screening tools in the pharmaceutical industry. Therefore, there is an urgent need in the industry for a feasible, accurate, and cost-effective solution that can efficiently integrate spatial information, enable visualized data association analysis, and be suitable for routine monitoring.

Geographic information systems (GIS) represent a fitting technological platform to address this challenge. GIS has been applied in various domains, such as identifying hotspots in soil microbial communities and monitoring disease transmission in public health, by leveraging its capabilities in spatial visualization, geospatial data extraction, and analytical functions to conduct research (16–20). Its core advantage lies in its capacity to precisely correlate complex data with geographical spatial locations and to accurately link microbial species with physical locations through spatial visualization and analysis techniques. This enables the identification of high-risk sites and potential pathways for contamination spread. Moreover, when considering cost-effectiveness, GIS is more economically efficient compared to WGS. Based on this, this study aimed to introduce GIS into the new scenario of environmental monitoring in the production of sterile drugs, not simply as a technological transplant, but rather to construct a methodology of “GIS-integrated strategy.” Through continuous collection and accurate identification (based on 16S rRNA/ITS) of environmental microorganisms in different cleanliness grades of sterile preparation workshop, and systematic integration and visualization of microbial data (types, quantity) and physical space (cleanliness grade, room layout, equipment location) of workshop with the help of GIS, we constructed a strategy for microbial contamination risk analysis in environmental monitoring, in order to realize the model transition from conventional “microbe presence” to “microorganism-environment-control” multi-dimensional correlation study.

The specific objectives of this study include (i) systematically analyzing the types, characteristics, and distribution patterns of microbial communities in the production environment of sterile pharmaceuticals; (ii) constructing a visualized environmental microbial map based on GIS to intuitively present the spatial distribution characteristics of microbes; (iii) integrating spatial visualization analysis with microbial contamination risk assessment strategies to identify key control areas and potential transmission pathways; validating the practical efficacy of this strategy in guiding precise contamination control through a specific intervention case. We anticipate that this work will provide a preliminary exploration for building a spatial cognition model of environmental microbes in pharmaceutical clean areas and offer a novel and effective scientific strategy for enhancing microbial control in aseptic pharmaceutical production.

## MATERIALS AND METHODS

### Main reagents

Tryptic soy agar medium (Guangzhou Baiyunshan Baidi Biopharmaceutical Co., Ltd.): used for the culture and counting of bacteria.

Sabouraud dextrose agar medium (Qingdao Rishui Biotechnology Co., Ltd.): used for the culture and counting of molds and yeasts.

### Main instruments

MiniCapt25M Airborne Microbe Collector (PMS Company, USA): used for active air sampling.

KB720 Constant Temperature Incubator (BINDER).

SeqStudio Flex Genetic Analyzer (Thermo Fisher Scientific).

Gradient 96-well Verti PCR Instrument (ABI, USA).

HR1200-11A2 (KY) Biosafety Cabinet (Haier).

### Collection and identification of environmental microorganisms

#### Environmental monitoring plan

This study was conducted within a GMP-certified production facility. Environmental monitoring was performed in accordance with pharmacopeial standards, with routine sampling locations determined through a grid-based method and/or hazard analysis and critical control point assessment. The monitoring program encompassed four environmental classifications: A grade (laminar airflow zone), B grade, C grade, and Controlled Not Classified (CNC) areas. Settle plates, active air sampling, and surface monitoring were employed in A, B, and C grade environments, whereas CNC areas were monitored using settle plates and surface sampling.

The sampling frequency was determined based on the environmental classification and risk assessment: A- and B-grade areas were sampled once per shift, C-grade areas were sampled once per week, and the CNC area was sampled once per quarter. All samples were cultured under standard conditions, and the isolated strains were subjected to 16S rRNA (for bacteria) or ITS (for fungi) gene sequence analysis to achieve strain identification at the species level.

#### Identification methods of environmental microorganisms

The molecular identification of strains involved a series of steps, including genomic DNA extraction, PCR amplification, product purification, and sequencing. DNA extraction was performed using a DNA extraction kit (TIANGEN BIOTECH [BEIJING] Co., LTD.) strictly following the manufacturer’s instructions. The PCR amplification primers used were universal primers 27f (AGAGTTTGATCCTGGCTCAG) and 1492r (TACGGCTACCTTGTTACGACTT) for bacterial identification, and ITS1 (TCCGTAGTTGAACCTGCGG) and ITS4 (TCCTCCGCTTATTGATATGC) for fungal identification.

Following isolation, bacterial strains were sent to the Hefei Customs District Technology Center for identification. The obtained sequences were submitted to the GenBank database for homology comparison. A sequence similarity threshold of ≥99% against type strains was used as the criterion for species-level identification (21), with all results formally documented in an identification report issued by the center.

### Statistical analysis

#### α and β diversity analysis

α diversity was evaluated using the Shannon index, which integrates both species richness and evenness within a community. This index is positively correlated with both richness and evenness, and it is used to characterize the level of microbial diversity within samples.

β diversity was measured using the Bray-Curtis distance, which quantifies the dissimilarity in microbial community composition between different samples. A larger distance value indicates a more significant difference in community structure between samples.

All statistical analyses were performed in the R environment (version 4.2.0). The α and β diversity analyses were conducted using the vegan package. The significance of differences between groups was assessed using the Kruskal-Wallis test for α diversity and PERMANOVA for β diversity. Data visualization was accomplished using the ggplot2 package, and community structure heatmaps were generated using the ComplexHeatmap package.

#### Contamination rate calculation

The contamination rate is defined as the proportion of the number of times microorganisms are detected in a specific area to the total number of monitoring events (22). The calculation formula is as follows:

Contamination rate (%) = (Number of monitoring events where microorganisms were detected/Total number of monitoring events) × 100%.

### Geographic information system

Although statistical analyses can help identify differences in microbial community composition, they have limitations in representing spatial patterns. To address this, spatial analysis and mapping were performed using ArcGIS (version 10.2). First, the architectural floor plan of the manufacturing facility was digitized and georeferenced to scale. The inverse distance weighting (IDW) interpolation method—a spatial prediction technique based on the principle of “spatial autocorrelation” (i.e., closer locations are more likely to have similar values than those farther apart)—was applied to model the spatial distribution of environmental microorganisms (23). In this approach, the influence of each sampled point decreases with increasing distance, allowing for continuous surface estimation of microbial abundance across the facility.

Second, establishing the association between microbial attribute data and spatial locations involves assigning microbial monitoring data containing strain identification results, quantitative counts, and sampling point identifiers to layer attribute tables. This data is then matched with corresponding sampling areas on controlled zone maps, and GIS visualization capabilities are employed to construct microbial maps with spatial referencing.

## RESULTS

### Environmental microbiota data

#### Distribution and quantity of environmental microorganisms

Between 2022 and 2025, through continuous environmental monitoring, this study collected a total of 1,117 microbial strains from different levels of clean areas. Following gene sequence analysis, species-level taxonomic identification was successfully obtained for all 1,117 bacterial isolates. Microbial composition analysis revealed that Gram-positive bacteria accounted for 73.3%, Gram-negative bacteria for 24.9%, and fungi for 1.8%. All fungi isolates were recovered exclusively from the CNC area. Specifically, 10 strains (0.9%) were isolated from A-grade clean areas, 77 strains (6.9%) from B-grade clean areas, 220 strains (19.7%) from C-grade clean areas, and 810 strains (72.5%) from the CNC area. The distribution of isolated strain counts across different cleanliness levels demonstrates that microbial contamination decreases significantly with increasing cleanliness level, consistent with the design expectations for gradient isolation between different cleanliness levels. Detailed data are presented in Table 1.

**TABLE 1:** Statistical analysis of environmental microorganisms across cleanliness grades

Cleanliness grade	Numbers of isolates	Species	Genus	Percentage (%)
A	10	9	5	0.9
B	77	29	15	6.9
C	220	55	23	19.7
CNC	810	173	57	72.5
Total	1,117	212	66	100

#### Distribution of A-grade environmental microorganisms

In the A-grade clean area environment, a total of 10 environmental microorganisms were isolated and identified, belonging to five different genera. Analysis of the genus composition revealed that the most abundant genus was *Staphylococcus* spp., accounting for 40.0%, followed by *Bacillus* spp. at 30.0%. *Kocuria* spp., *Moraxella* spp., and *Micrococcus* spp. each accounted for 10.0%. The microbial community analysis indicated that Gram-positive bacteria constituted 90.0%, while Gram-negative bacteria made up 10.0%. In terms of sampling sources, strains obtained from settle plates accounted for 70.0%, while those from surface samples accounted for 30.0%. Detailed data are presented in Table 2.

**TABLE 2:** Identification results of A-grade environmental microorganisms

No.	Genus	Species	Isolates	Air sample	Settle plates	Surface sample	Gram-positive/Gram-negative
1	*Staphylococcus*	*Staphylococcus cohnii*	2	0	2	0	Gram-positive
		*Staphylococcus hominis*	1	0	1	0	
		*Staphylococcus caprae*	1	0	1	0	
2	*Bacillus*	*Bacillus pacificus*	1	0	0	1	Gram-positive
		*Bacillus cereus*	1	0	0	1	
		*Bacillus amyloliquefaciens*	1	0	0	1	
3	*Kocuria*	*Kocuria rhizophila*	1	0	1	0	Gram-positive
4	*Moraxella*	*Moraxella osloensis*	1	0	1	0	Gram-negative
5	*Micrococcus*	*Micrococcus luteus*	1	0	1	0	Gram-positive

#### Distribution of B-grade environmental microorganisms

In the B-grade clean area environment, a total of 77 environmental microorganisms were isolated and identified, belonging to 15 different genera. Analysis of the genus distribution revealed that *Staphylococcus* spp. was the dominant genus, accounting for 41.6%, followed by *Micrococcus* spp. at 24.7% and *Bacillus* spp. at 14.3%. Additionally, *Corynebacterium* spp. and *Kocuria* spp. each accounted for 2.6%, while the remaining 10 genera collectively accounted for 14.2%. The microbial community analysis indicated that Gram-positive bacteria were overwhelmingly dominant (93.5%), while Gram-negative bacteria accounted for only 6.5%. In terms of strain source composition, surface samples had the highest proportion (61.0%), followed by air samples (22.1%) and settle plates (16.9%). Detailed data are presented in Table 3.

**TABLE 3:** Identification results of B-grade environmental microorganisms

No.	Genus	Species^*a*^	Isolates	Air sample	Settle plates	Surface sample	Gram-positive/Gram-negative
1	*Staphylococcus*	*Staphylococcus epidermidis*	8	0	0	8	Gram-positive
		*Staphylococcus hominis*	8	1	3	4	
		*Staphylococcus capitis*	6	1	1	4	
2	*Micrococcus*	*Micrococcus luteus*	19	5	6	8	Gram-positive
3	*Bacillus*	*Bacillus cereus*	5	0	0	5	Gram-positive
		*Lysinibacillus halotolerans*	2	0	0	2	
		*Bacillus aryabhattai*	1	0	0	1	
4	*Corynebacterium*	*Corynebacterium sanguinis*	1	1	0	0	Gram-positive
		*Corynebacterium glutamicum*	1	1	0	0	
5	*Kocuria*	*Kocuria rhizophila*	1	0	0	1	Gram-positive
		*Kocuria palustris*	1	0	0	1	
6	Other	N/A	11	1	2	8	N/A^*b*^

^*a*^ This table presents only the top three most frequently isolated species from each genus as representative examples.

^*b*^ N/A = not available.

#### Distribution of C-grade environmental microorganisms

In the C-grade clean area environment, a total of 220 environmental microorganisms were isolated and identified, belonging to 23 different genera. Analysis of the genus distribution revealed that *Staphylococcus* spp. was the dominant genus, accounting for 54.5%, followed by *Micrococcus* spp. at 13.6%. *Kocuria* spp. and *Sphingomonas* spp. each accounted for 4.5%, while *Moraxella* spp. accounted for 4.1%. The remaining 18 genera collectively accounted for 18.8%. The microbial community analysis indicated that Gram-positive bacteria constituted 79.1%, while Gram-negative bacteria accounted for 20.9%. In terms of strain source composition, surface samples had the highest proportion (49.1%), followed by settle plates (34.1%) and air samples (16.8%). Detailed data are presented in Table 4.

**TABLE 4:** Identification results of C-grade environmental microorganisms^*a*^

No.	Genus	Species	Isolates	Air sample	Settle plates	Surface sample	Gram-positive/Gram-negative
1	*Staphylococcus*	*Staphylococcus epidermidis*	37	7	9	21	Gram-positive
		*Staphylococcus warneri*	23	2	4	17	
		*Staphylococcus hominis*	16	2	4	10	
2	*Micrococcus*	*Micrococcus luteus*	28	2	10	16	Gram-positive
		*Micrococcus cohnii*	1	1	0	0	
		*Micrococcus antarcticus*	1	1	0	0	
3	*Kocuria*	*Kocuria rhizophila*	2	1	1	0	Gram-positive
		*Kocuria palustris*	2	1	0	1	
		*Kocuria kristinae*	2	0	1	1	
4	*Sphingomonas*	*Sphingomonas paucimobilis*	10	5	3	2	Gram-negative
5	*Moraxella*	*Moraxella osloensis*	9	1	7	1	Gram-negative
6	Other	N/A	41	10	17	14	N/A^*b*^

^*a*^ This table presents only the top three most frequently isolated species from each genus as representative examples.

^*b*^ N/A = not available.

#### Distribution of CNC area environmental microorganisms

In the CNC area environment, a total of 810 environmental microorganisms were isolated and identified, representing 57 different genera. Analysis of the genus distribution revealed that *Staphylococcus* spp. was the most abundant genus, accounting for 31.4%, followed by *Micrococcus* spp. at 13.1%, *Bacillus* spp. at 11.9%, *Acinetobacter* spp. at 5.7%, and *Sphingomonas* spp. at 4.8%. The remaining 52 genera collectively accounted for 33.1%.

Further analysis indicated that in the CNC area environment, Gram-positive bacteria constituted 70.0%, Gram-negative bacteria accounted for 27.5%, and fungi accounted for 2.5%. In terms of strain source composition, surface samples were the predominant source, representing 89.0%, while settle plates accounted for 11.0%. Detailed data are presented in Table 5.

**TABLE 5:** Identification results of CNC area environmental microorganisms^*a*^

No.	Genus	Species	Isolates	Settle plates	Surface sample	Gram-positive/Gram-negative
1	*Staphylococcus*	*Staphylococcus warneri*	52	11	41	Gram-positive
		*Staphylococcus epidermidis*	51	6	45	
		*Staphylococcus hominis*	44	6	38	
2	*Micrococcus*	*Micrococcus luteus*	103	4	99	Gram-positive
		*Micrococcus lylae*	2	1	1	
3	*Bacillus*	*Paenibacillus glucanolyticus*	19	14	5	Gram-positive
		*Bacillus cereus*	15	1	14	
		*Bacillus licheniformis*	8	0	8	
4	*Acinetobacter*	*Acinetobacter johnsonii*	23	0	23	Gram-negative
		*Acinetobacter lwoffii*	18	0	18	
		*Acinetobacter radioresistens*	3	0	3	
5	*Sphingomonas*	*Sphingomonas paucimobilis*	24	2	22	Gram-negative
		*Sphingomonas dokdonensis*	4	1	3	
		*Sphingobium yanoikuyae*	3	0	3	
6	Fungi	N/A	20	4	16	N/A
7	Other	N/A	248	18	230	N/A^*b*^

^*a*^ This table presents only the top three most frequently isolated species from each genus as representative examples.

^*b*^ N/A = not available.

#### Comparison of microbial abundance across cleanroom grades

**Fig 1:**
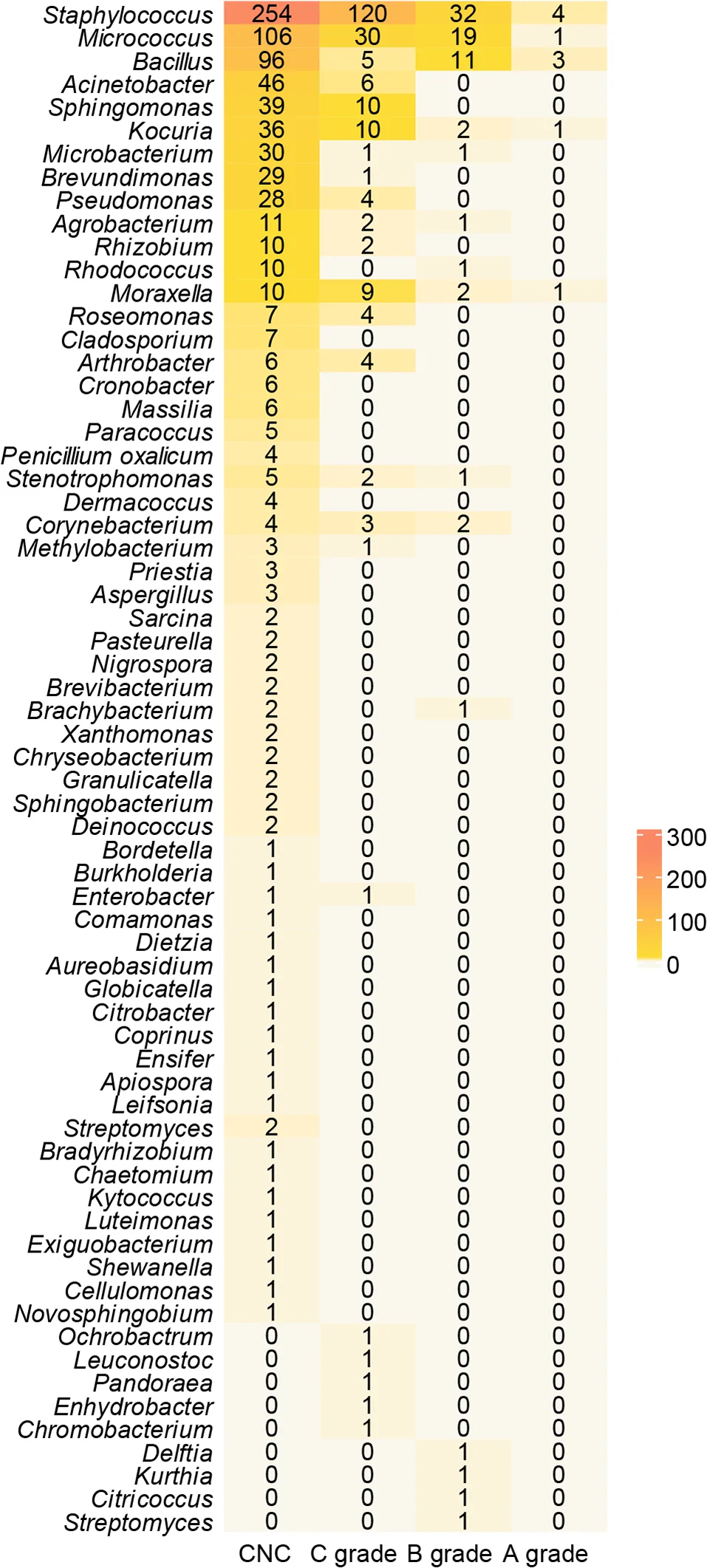
Heatmap of environmental microbiota abundance.

A comparative analysis of the abundance of environmental microorganisms in the CNC area and A, B, and C grades clean areas of the production workshop was conducted at the genus level. The heatmap results clearly demonstrate (see Fig. 1) that the microbial community structures of these areas are significantly different, with microbial contamination decreasing in a gradient manner as the clean level increases. The CNC area has a significantly higher abundance of environmental microorganisms than the other areas, with a total of 57 genera of microorganisms detected, including fungi, indicating high microbial diversity and complex ecological structure in this area. The abundance of bacterial genera decreases sequentially from C-grade to B-grade areas, with 23 and 15 genera detected, respectively. A-grade area has the fewest types of bacterial genera (only five genera) and the lowest abundance, with no fungi detected.

#### Analysis of microbial diversity across cleanroom grades

This study analyzed the diversity of microbial community structures in the CNC area and the A, B, and C grade cleanrooms of a Sterile Pharmaceutical, based on species-level taxonomic identification.

**Fig 2:**
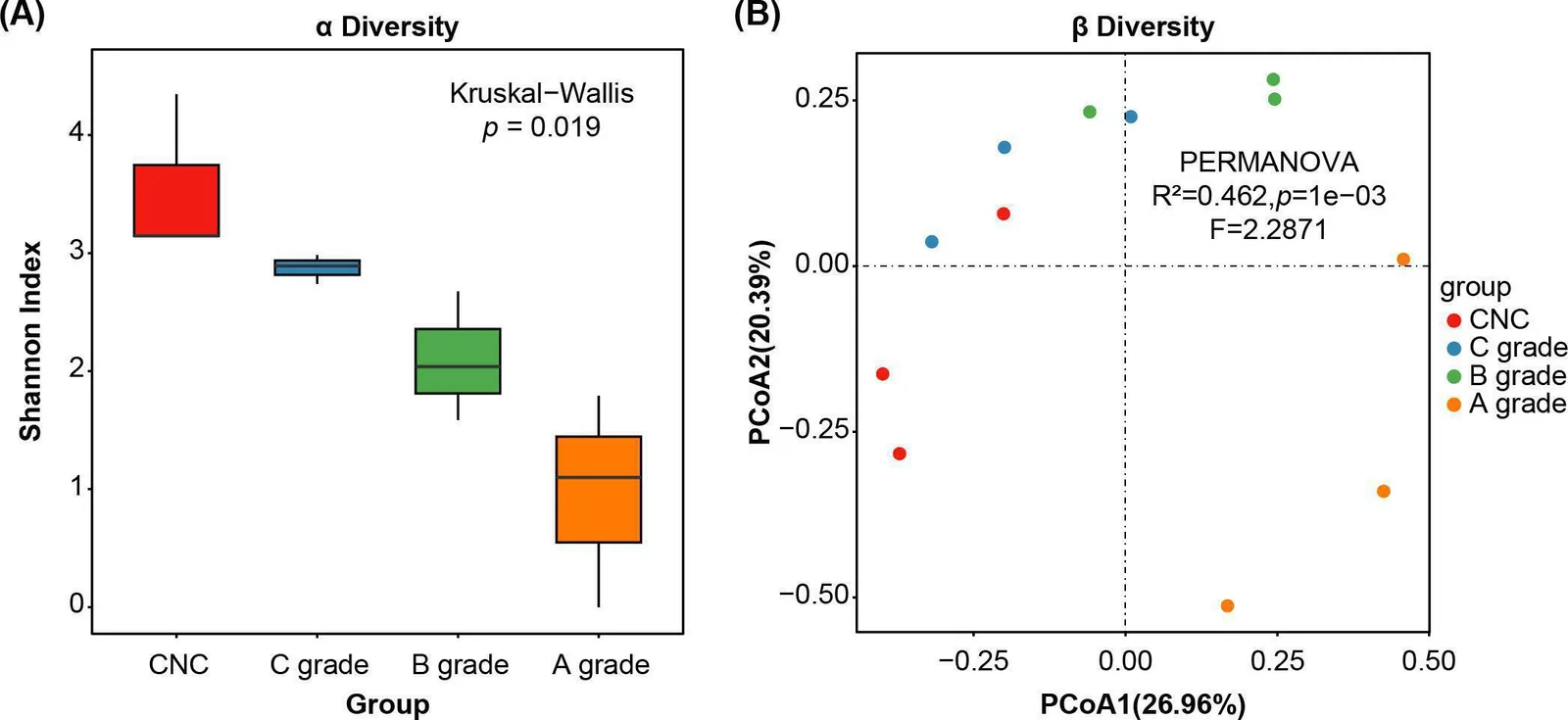
Analysis of environmental microbial diversity.(**A**) Analysis of microbial α diversity within samples; (**B**) Analysis of β diversity based on Bray-Curtis distance.

The Shannon index was employed to assess the microbial α-diversity within samples. The CNC area exhibited a broad range of Shannon index values, with the highest value reaching 4.35, indicating a high level of species diversity in this group of samples. The Shannon index in C grade was relatively concentrated, ranging from 2.74 to 2.98, representing a moderate level of diversity. B grade had a Shannon index distribution ranging from 1.59 to 2.68, indicating a lower level of diversity. A grade had the narrowest range of Shannon index values, between 1.10 and 1.79, indicating the lowest level of diversity. According to the results of the Kruskal-Wallis test, there were significant overall differences in the Shannon index values among the four groups of samples (*P* = 0.019). See Fig. 2A for details.

β-diversity analysis was based on Bray-Curtis distance, which was used to assess the similarity of microbial community composition between samples and was visualized through principal coordinates analysis (PCoA). The PCoA results showed that A-grade clean area had a larger Bray-Curtis distance (0.89–0.99) compared with the other three groups (CNC, C, and B), indicating a significant difference in community structure from the other levels. In contrast, the community distances between the CNC area, C grade, and B grade were relatively smaller (0.54–0.95), suggesting a greater similarity in community structure among these three groups. PERMANOVA analysis further confirmed that the factor of “clean level” had a significant impact on the variation of microbial community structure (R² = 0.462, *P* = 0.001, F = 2.2871), indicating that the differences in community structure among different clean levels were statistically significant. See Fig. 2B for details.

#### Comparison of environmental isolates by sampling source

**Fig 3:**
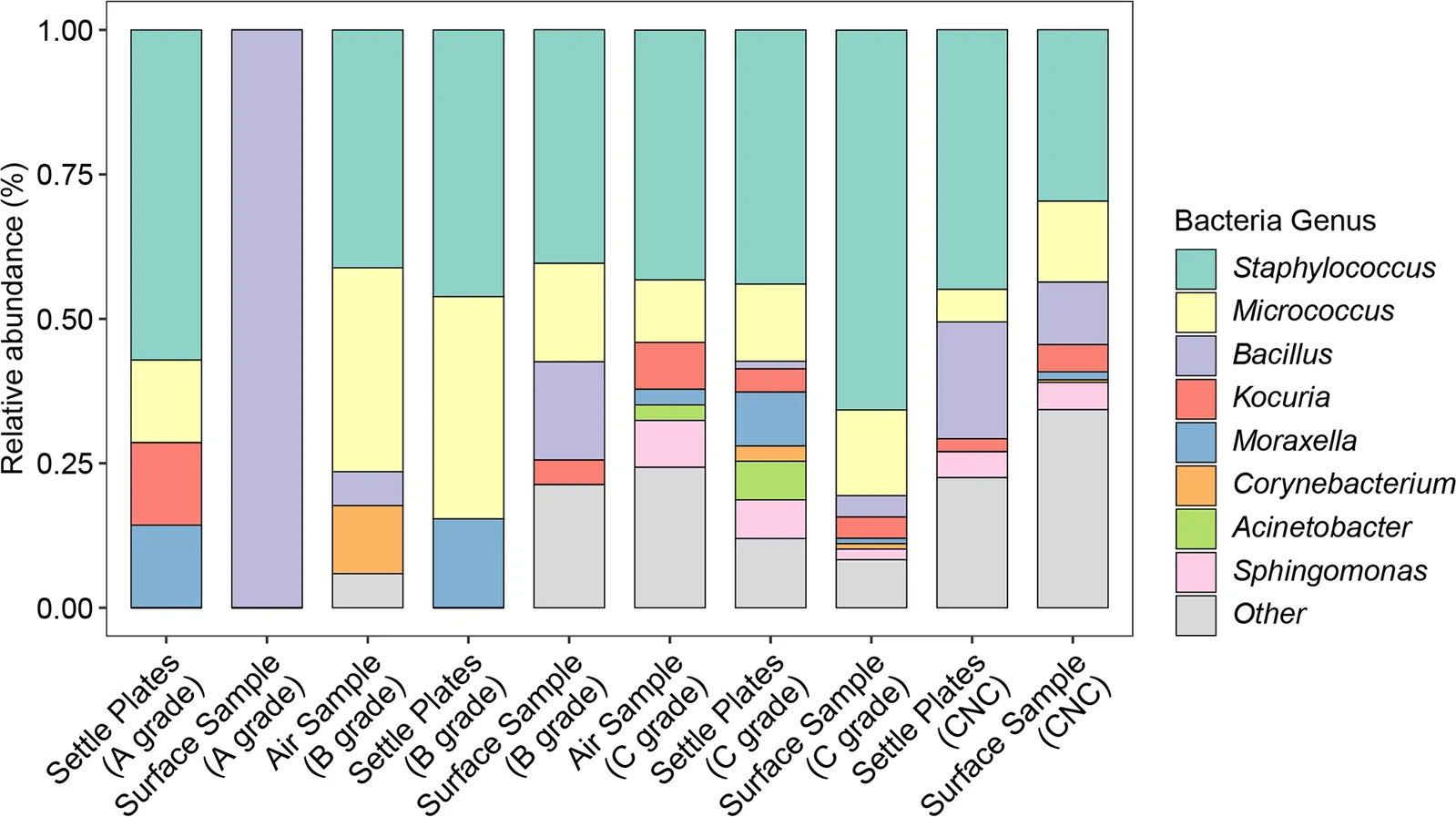
Comparison of genus-level distribution of environmental isolates by sampling source.

Comparison of the genus-level distribution of environmental microorganisms (air samples, settle plates, and surface samples) in the CNC area and A, B, and C grades clean areas (Fig. 3) revealed that *Staphylococcus* was the most widely distributed core microbial genus across all environments. This genus dominated in all types of samples, with particularly high relative abundance in A grade settle plates (57.1%), B grade air samples (41.2%), settle plates (46.2%), and surface samples (40.4%), C grade air samples (43.2%), settle plates (44.0%), and surface samples (65.7%), as well as in CNC area settle plates (44.9%) and surface samples (29.7%), significantly higher than other genera. *Micrococcus* was the second most dominant genus, widely present in air samples, settle plates, and surface samples, with a distribution breadth second only to *Staphylococcus*. Additionally, *Bacillus* was detected in all clean levels, particularly in surface samples of A and B grade environments.

#### Characterization of environmental microbial isolates

**Fig 4:**
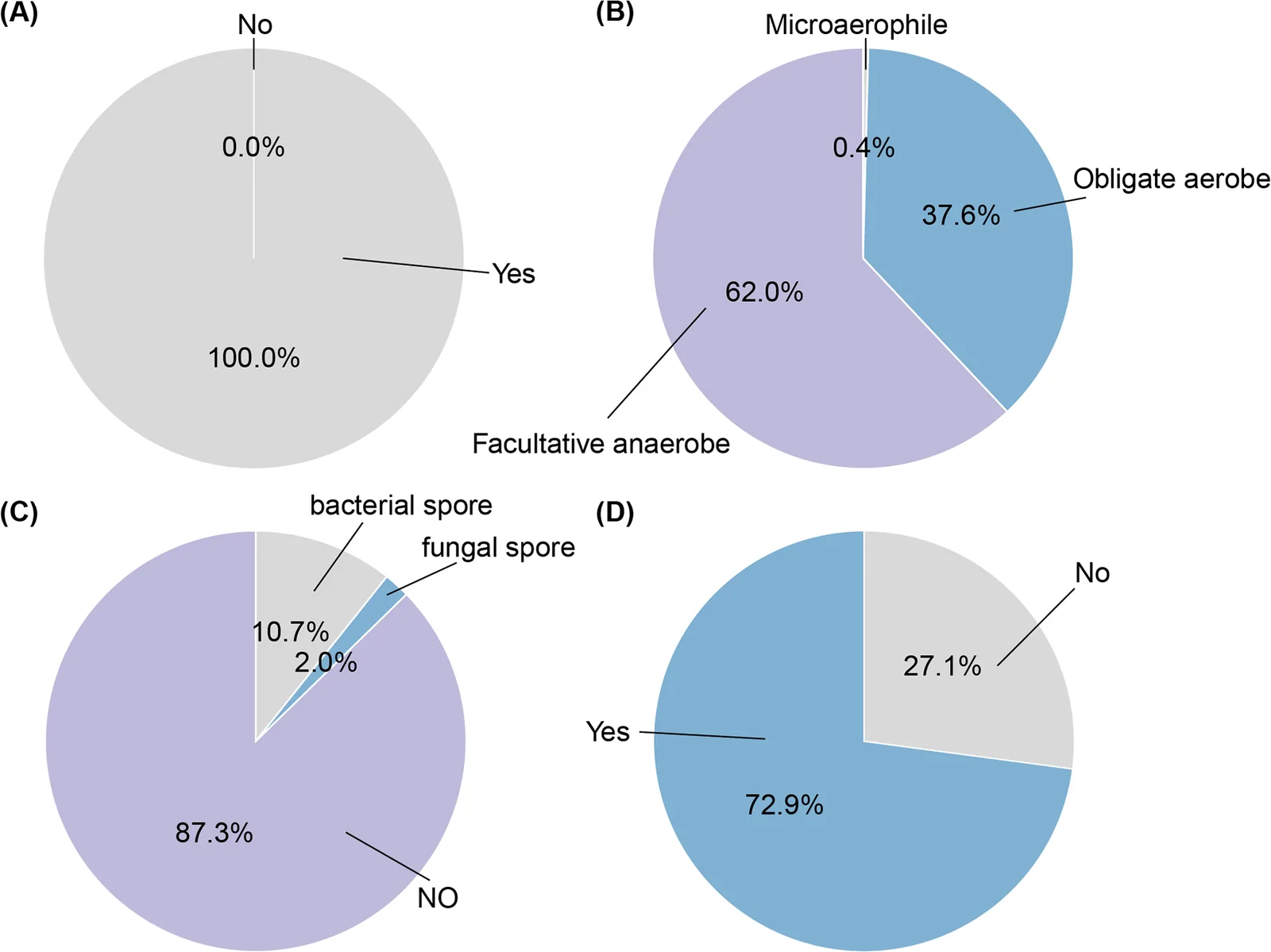
Characterization of environmental microbial isolates.(**A**) Biofilm-forming ability; (**B**) Aerobic type; (**C**) Sporulation ability; (**D**) Motility).

Characterization analysis was conducted on the environmental microorganisms isolated in this study. The results indicated that all isolated strains possessed the capability to form biofilms (Fig. 4A), suggesting the potential for environmental microorganisms to form biofilms on equipment surfaces and the inner walls of pipelines. Analysis of aerobic types revealed that facultative anaerobes (62.0%) were the dominant group, followed by obligate aerobes (37.6%), with microaerophiles (0.4%) accounting for a very low proportion (Fig. 4B). Moreover, the vast majority of strains (87.3%) did not produce spores, which is conducive to the effectiveness of routine disinfection measures. However, 12.7% of the strains were capable of forming highly resistant spores (10.7%) or fungal spores (2.0%), which exhibit strong tolerance to moist heat, radiation, and chemical disinfectants. Regarding motility, 72.9% of the strains possessed flagella (polar or peritrichous) that mediated motility. In summary, the environmental microorganismal reservoir in this study area is primarily composed of strains that possess biofilm-forming ability, motility, and facultative anaerobic metabolism.

### Microbial mapping, risk analysis, and contamination control

#### Visual analysis of environmental microbiota data

The effectiveness of contamination control strategies relies on the accurate identification of contamination sources. In this case study, focusing on C-grade clean area, we utilized GIS to construct a visualized spatial distribution map of environmental microorganisms. By comparing the changes in microbial communities before and after the implementation of control measures, we analyzed the actual effectiveness of the intervention strategies.

**Fig 5:**
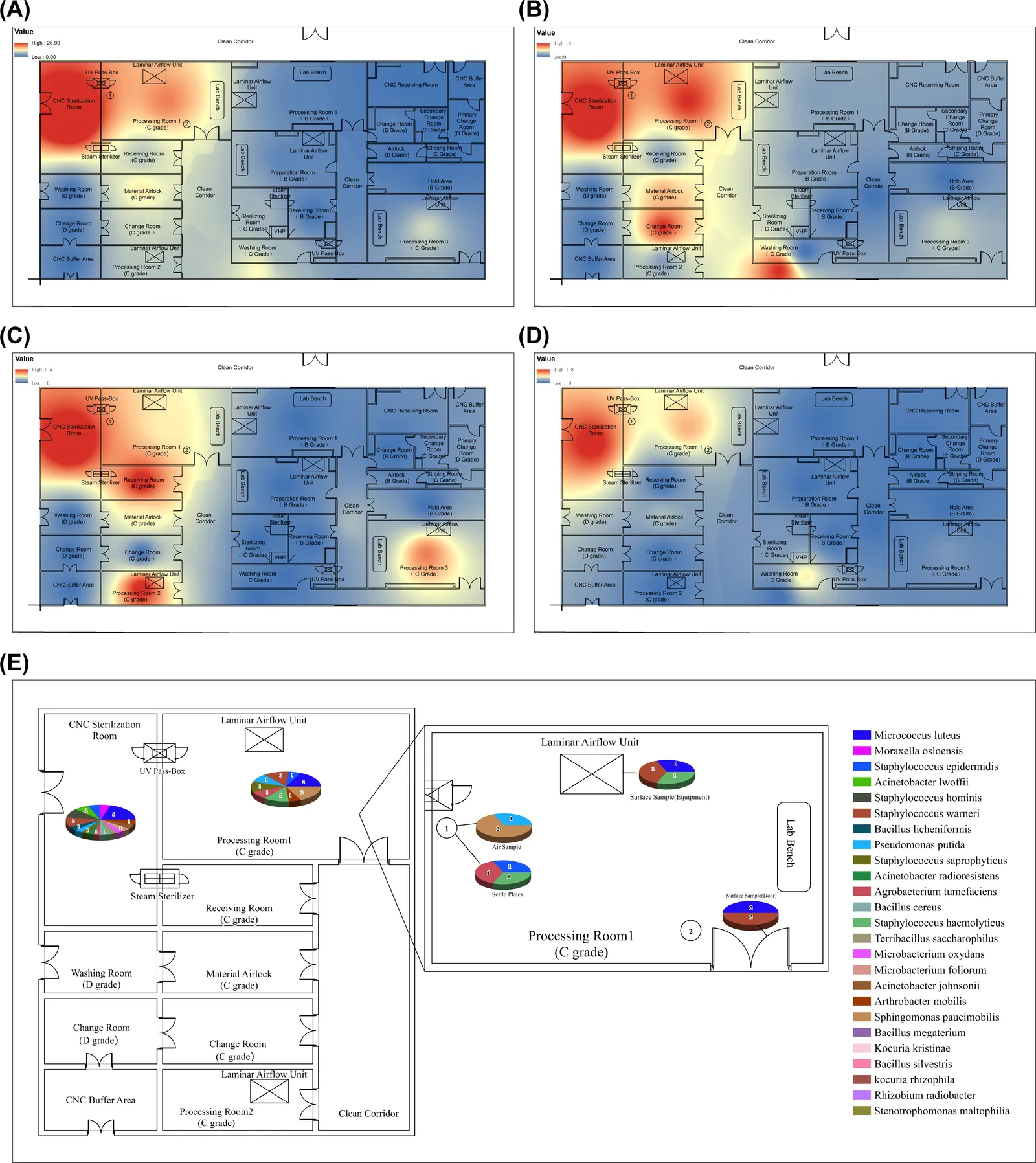
Spatial distribution of environmental microbiota (I). (**A**) Overall microbial distribution hotspot map; (**B**) Staphylococcus; (**C**) Micrococcus; (**D**) non-human-source microorganisms; (**E**) microbial distribution characteristics map.

Based on the IDW visualization analysis, it can be observed that the hotspot areas with relatively high absolute abundance of environmental microorganisms in phase I are mainly distributed in the CNC sterilization room and C-grade Processing Room 1 (Fig. 5A). During this phase, items that cannot be sterilized by moist heat enter the C-grade clean area Processing Room 1 through airlocks and UV pass-box. A total of 27 strains of environmental microorganisms were identified in the CNC sterilization room, while 13 strains were identified in C-grade clean area Processing Room 1.

Human-associated microorganisms (such as *Staphylococcus* and *Micrococcus*) in the C-grade clean area are widely distributed, with hotspot areas of higher absolute abundance found across various processing rooms in the C-grade environment (Fig. 5B and C). Non-human-source microorganisms (such as *Pseudomonas* and *Agrobacterium*) are primarily distributed in three areas: the CNC sterilization room, C-grade clean area, Processing Room 1, and the washing room (Fig. 5D. The frequency of microorganisms detected in the B-grade clean area is relatively low, and all identified microorganisms belong to *Staphylococcus* and *Micrococcus*.

Further GIS-based visualization of microbial distribution revealed that *Pseudomonas putida* and *Agrobacterium tumefaciens* isolated from site 1 in C-grade processing room, as well as *Arthrobacter agilis* from equipment surface microorganisms, were also detected in adjacent CNC areas (Fig. 5E).

#### Strategy for microbial contamination risk analysis

Spatial configuration analysis confirmed that the UV pass-box constituted the shortest path (approximately 1 m) between the CNC area and the C-grade cleanroom. Microbial community analysis revealed that the CNC sterilization room and adjacent C-grade Processing Room 1 exhibited relatively high microbial abundance with overlapping community structures, particularly in non-human-source environmental microorganisms, such as *Pseudomonas putida* and *Agrobacterium tumefaciens*, with *Pseudomonas putida* having a strong biofilm-forming ability. Additionally, human-associated microorganisms, such as *Staphylococcus* and *Micrococcus,* were detected within the C-grade Processing Room 1. The Bray-Curtis distance between the two environmental samples was 0.50, further supporting the high similarity in community structure and suggesting potential vulnerabilities in material transfer (via the UV pass-box) and personnel movement pathways that could lead to cross-contamination.

**Fig 6:**
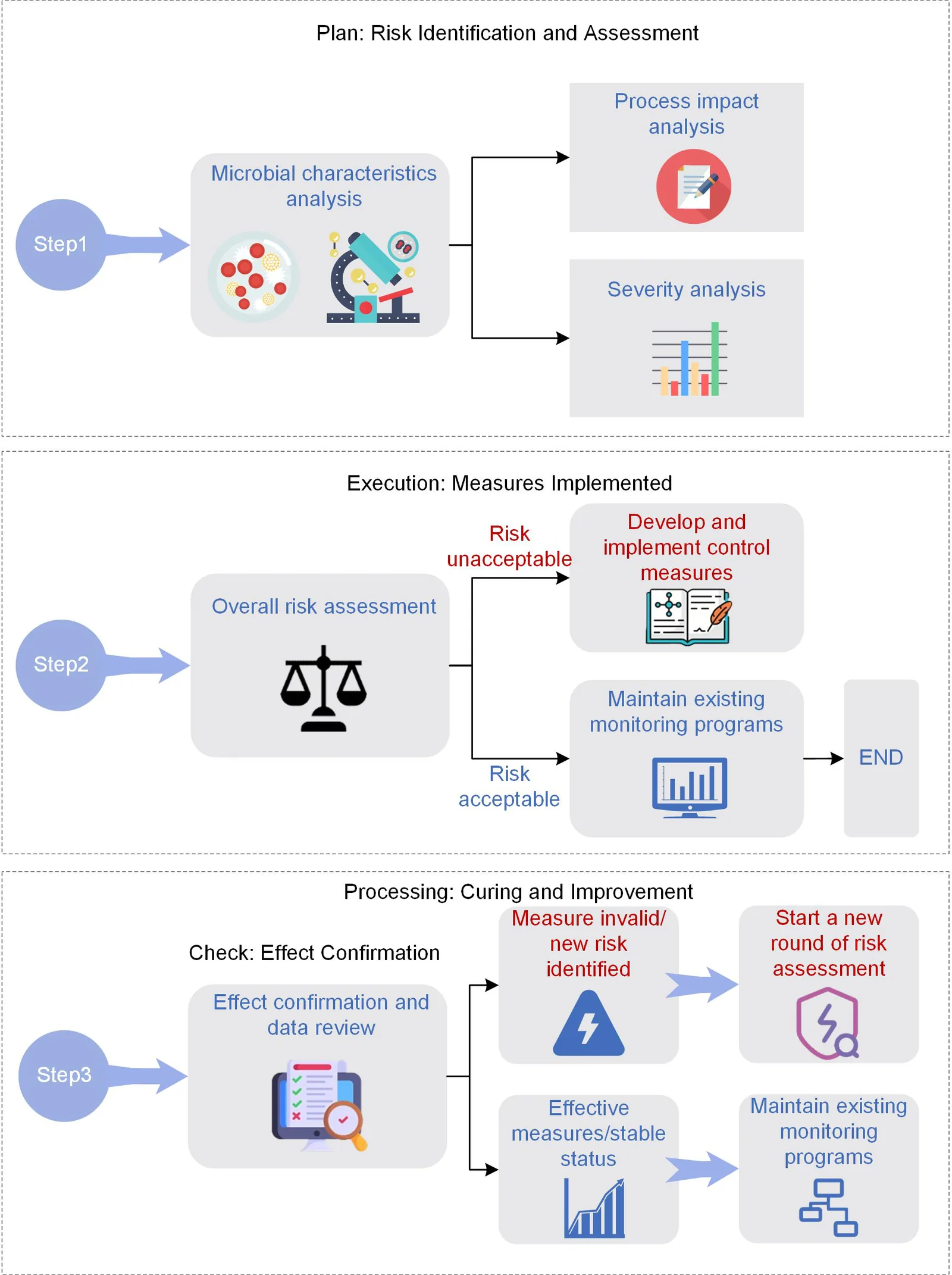
Strategy for microbial contamination risk analysis. This strategy establishes a comprehensive microbial risk management system. First, risks are identified through microbial characterization (e.g., species identification, biofilm formation capacity), followed by root cause and contamination pathway analysis. The severity is then assessed based on process characteristics. A holistic risk evaluation is conducted by integrating multidimensional biological features and process analysis outcomes. If the risk is deemed unacceptable, corresponding control measures are planned and implemented, the effectiveness of which is subsequently verified through comparative monitoring data. Ultimately, effective solutions are institutionalized based on results, or a new round of assessment is initiated, thereby forming a closed-loop PDCA (Plan-Do-Check-Act) management cycle.

In this study, based on the understanding of process knowledge, a microbial contamination risk analysis strategy, as shown in Fig. 6, was formulated. In accordance with this strategy, closed-loop contamination risk analysis and control were implemented. First, the characteristics of the strains (such as spore formation capacity, biofilm formation ability, etc.) and their correlations with spatial distribution were analyzed to identify contamination pathways and their potential impact on product safety. Subsequently, a multi-dimensional impact assessment was conducted in combination with the production process to determine whether control measures needed to be taken. The analysis results indicate that C-grade Processing Room 1, as a partial production area for cell-based biologics, employs an open process, and the existing contamination risk is unacceptable. Therefore, it is necessary to develop targeted contamination control measures for the material transfer pathways to reduce the risk of cross-contamination.

#### Implementation of contamination controls

To enhance the level of microbial contamination control, this study implemented targeted measures based on the identified contamination pathways: the risky UV transfer method was eliminated to block the pathway for environmental microorganisms transmission from the CNC area; subsequently, items were uniformly transferred into the clean area via the airlock passage, during which sporicidal treatment was applied, and room pressure control was improved.

**Fig 7:**
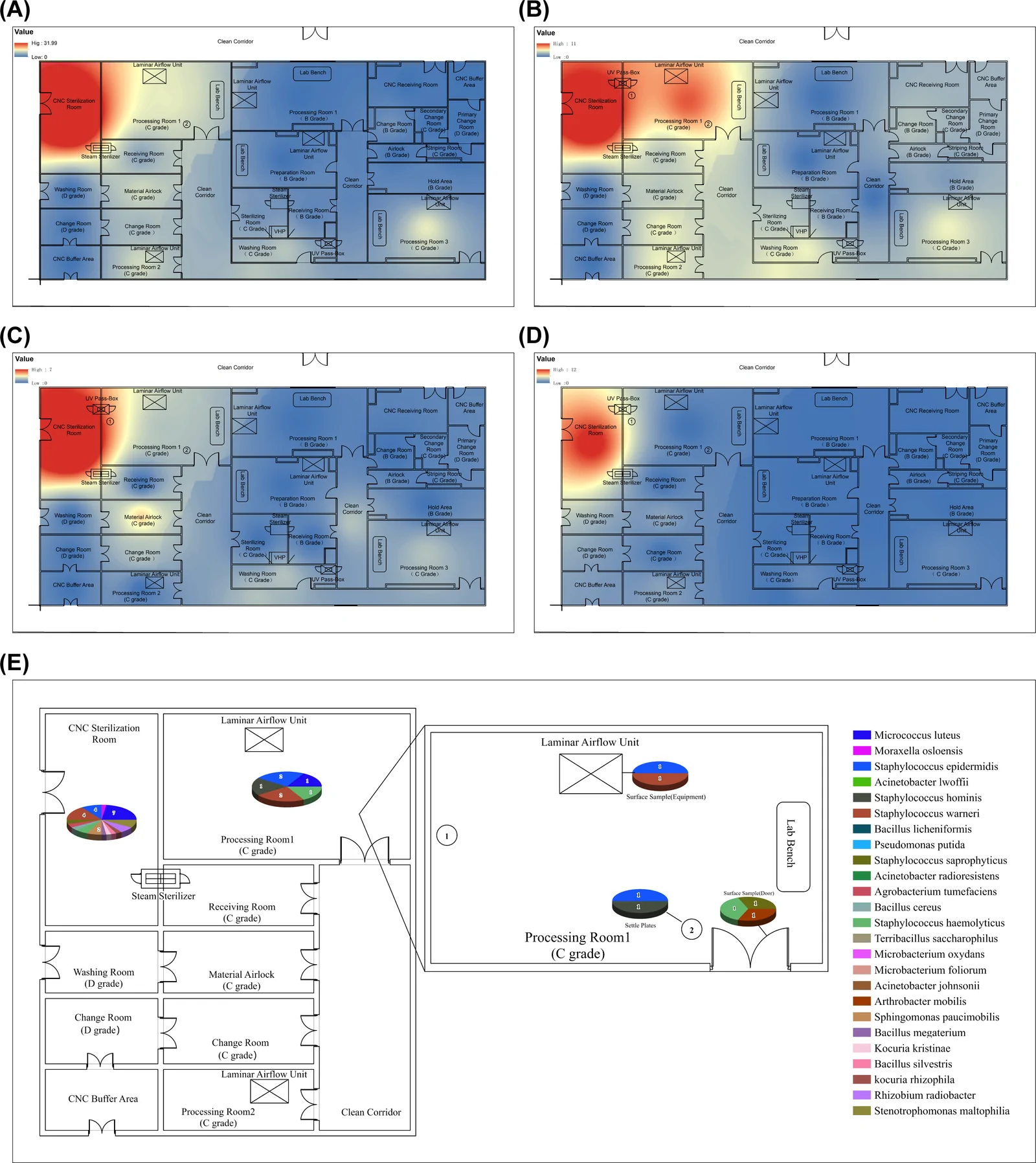
Spatial distribution of environmental microbiota (II). (**A**) Overall microbial distribution hotspot map; (**B**) Staphylococcus; (**C**) Micrococcus; (**D**) non-human-source microorganisms; (**E**) microbial distribution characteristics map.

To investigate the efficacy of control measures and variations in microbial distribution, visualization analysis was performed in the target area using the IDW method. The results indicated that the CNC sterilization room remained a hotspot with relatively high absolute bacterial abundance, whereas a substantial reduction in microbial abundance was observed in C-grade Processing Room 1 (Fig. 7A). A total of 32 environmental strains were identified in the CNC sterilization room, in contrast to only seven strains detected in the adjacent C-grade Processing Room 1, all of which belonged to the *Staphylococcus* and *Micrococcus* genera.

In the C-grade clean area, locations with high absolute abundance of human-associated microorganisms (such as *Staphylococcus* and *Micrococcus*) remained concentrated in the processing rooms (Fig. 7B and C), whereas no non-human-associated microorganisms were detected in C-grade Processing Room 1, though they were still found in the material airlock and washing room (Fig. 7D).

GIS-based visualization of microbial distribution further showed that neither airborne nor settling bacteria were detected at the previously identified high-risk site (site 1) (Fig. 7E). Beta-diversity analysis of environmental samples from the two locations revealed a Bray-Curtis distance of 0.69, indicating a significant increase in the structural dissimilarity between the microbial communities of the two areas.

Visual and diversity analyses comparing environmental flora distribution across two phases demonstrate the effectiveness of the implemented control measures. Further analysis of contamination rates in C-grade Processing Room 1 before and after intervention shows a reduction from 4.3% to 2.2%. Collectively, these data indicate that the targeted comprehensive control measures effectively disrupted microbial transmission from the CNC area to the clean zone. A detailed comparison between the two phases is presented in Table 6.

**TABLE 6:** Comparative analysis of compositional differences in environmental microbiota

Different stages	C grade room contamination rate (%)	β diversity (Bray-Curtis)	CNC area and grade C processing room co-existing environmental microorganisms	Contamination source analysis
Phase I	4.3	0.5	*Staphylococcus haemolyticus* *Staphylococcus warneri* *Staphylococcus epidermidis* *Staphylococcus saprophyticus* *Micrococcus luteus*	Personnel
			*Agrobacterium tumefaciens* *Arthrobacter mobilis*	Soil
			*Pseudomonas putida*	Water source, soil
Phase II	2.2	0.69	*Staphylococcus epidermidis* *Staphylococcus warneri* *Staphylococcus haemolyticus* *Micrococcus luteus*	Personnel

## DISCUSSION

### Distribution characteristics of environmental microorganisms

Analysis of environmental monitoring results in the sterile formulation production workshop from 2022 to 2025 revealed that the CNC area had the highest abundance of environmental microorganisms, with diverse species and complex ecology. Compared to the CNC area, the microbial contamination levels in A, B, and C grades clean areas decreased progressively, demonstrating distinct purification characteristics. This indicates that the comprehensive measures, including personnel and material management and environmental disinfection, implemented in the clean areas are effective in controlling microbial contamination. *Staphylococcus* and *Micrococcus* were the predominant environmental microorganisms in all areas, consistent with previous studies (6, 7), further confirming that these two genera are primarily sourced from human contamination. Notably, *Corynebacterium*, *Sphingomonas*, and *Kocuria* were found more frequently in the clean areas, suggesting that the distribution of environmental microorganisms in clean areas is specific to geographical location and microbial control measures (13, 24).

The results of this study also indicate that the distribution of environmental microorganisms in adjacent clean areas is correlated, with microbial communities showing a pattern of continuous succession. This suggests that environmental microorganisms from the CNC area may enter the clean areas through human and material traffic, indicating that microbial contamination control should not only block or control transmission pathways but also focus on and manage contamination sources. Effective management of microorganisms in the CNC area of the production workshop can further enhance the effectiveness of microbial control in clean areas. The microbial distribution in A-grade clean areas shows the weakest correlation with other clean area levels and no clear succession pattern in genus occurrence. This phenomenon suggests that the extreme physical and chemical conditions of A-grade environments (such as unidirectional airflow and high-frequency disinfection) create strong selective pressures. These pressures lead to a simplified and stochastic microbial community structure, thereby validating the effectiveness of existing environmental control measures.

Based on the analysis of the biological characteristics of environmental microorganisms, the microbial strains that can be isolated in pharmaceutical clean environments constitute a reservoir with multiple adaptive features, including surface colonization (biofilm formation), environmental adaptation (facultative anaerobiosis), resistance mechanisms (spore formation), and active dispersal (motility). These characteristics enable these microorganisms to continuously withstand routine cleaning and disinfection measures, posing a persistent challenge to clean area environments. Therefore, integrating environmental monitoring data with spatial analysis is particularly important, as it helps identify potential colonization sites and transmission pathways of high-risk bacteria, thereby promoting a shift in environmental control strategies from “reactive response” to “proactive early warning.” Moreover, the goal of environmental monitoring should not merely be to “identify species,” but also to “assess their functional traits,” as these traits directly determine the level of contamination risk and inform the formulation of control strategies.

### The significance of GIS integration analysis in environmental microorganisms research

The current environmental monitoring in the pharmaceutical industry has two major shortcomings. First, there is an overemphasis on microbial counts while neglecting the risk information contained in species characteristics and spatial distribution. Second, there is a lack of a systematic microbial contamination risk analysis strategy, which leads to companies resorting to indiscriminate large-scale cleaning and disinfection when facing environmental contamination issues. This approach is not only costly and inefficient but also fails to fundamentally eliminate contamination.

This study, based on the “microorganism-environment-control” theoretical framework, incorporates GIS technology to analyze the spatial distribution characteristics of environmental microorganisms in pharmaceutical facilities. It successfully generated hotspot distribution maps of total environmental microbes, as well as hotspot distribution maps and spatial characteristic diagrams for different microbial groups, thereby revealing microbial distribution patterns. This approach achieves the visualization of microbial contamination distribution and can serve as an indicator for assessing environmental conditions. On the basis of spatial distribution analysis, the study integrates microbial contamination analysis strategies (multidimensional analysis and control strategies, including microbial characteristic analysis, biodiversity analysis, identification of process characteristics, and contamination rates). This assists in identifying contamination sources, provides a basis for enterprises to formulate scientifically targeted intervention measures, and facilitates the implementation of precise interventions. Case study results show that GIS integrated analysis can enhance scientific understanding of microbial distribution, support contamination traceability, and the implementation and evaluation of targeted control. Thus, it provides scientific and refined data support for the formulation of contamination control strategies in sterile drug production, avoiding resource waste caused by indiscriminate large-scale cleaning.

The core value of this strategy lies in elevating environmental microbial monitoring from isolated “data point” analysis to an integrated “spatial data + microbial attribute” analytical framework. By synthesizing microbial diversity analysis with spatial distribution characteristics, microbial information can more effectively guide contamination control. The specific advantages are manifested in three aspects: first, it identifies microbial spatial distribution patterns that are difficult to capture with traditional methods and reveals the intrinsic relationships between species distribution and environmental factors, such as clean area layout and equipment location. Second, it promotes a shift in environmental monitoring from “focusing solely on species presence” to a dynamic management model of “microorganism-environment-control.” Third, it demonstrates good cost controllability, real-time response capability, and decision support efficiency in routine monitoring, making it more suitable for daily risk screening and trend monitoring. In contrast, WGS is more appropriate for in-depth source tracing of specific deviations or contamination events (25).

### Strategies for mapping environmental microorganisms

Spatial analysis, as a core function of GIS, provides an effective tool for understanding the distribution patterns, transmission pathways, and potential sources of environmental microorganisms. Its strength lies in the ability to integrate multiple layers of spatial data (such as environmental and temporal parameters) and to conduct spatial analysis and modeling, thereby enabling in-depth interpretation of microbial distribution patterns (26).

Despite the phased achievements obtained through the application of GIS in this study, several limitations remain, which also point the way for future research:

First, the current analysis primarily relies on the spatial correlation characteristics of microbial distribution and has not yet incorporated real-time environmental parameters (such as particle counts, temperature, and humidity) as covariates for integrated analysis. This limitation constrains our ability to gain deeper insights into the influence of specific environmental factors on the spatial distribution of microorganisms. A potential confounding factor is that variations in microbial abundance driven by environmental fluctuations—such as localized anomalies in particle counts—may be incorrectly attributed entirely to specific personnel or material pathways. This could compromise the absolute accuracy of contamination source identification.

Second, the current sampling strategy, which is based on conventional environmental monitoring grids, fails to capture areas where microorganisms may potentially colonize and persist (e.g., internal crevices of equipment, high-efficiency particulate air supply outlets, and exhaust vents). This may result in an incomplete “contamination hotspot map,” potentially underestimating risks associated with hard-to-access microbial reservoirs. Incorporating a spatially randomized sampling approach in future work would provide complementary data, enabling a more comprehensive representation of microbial distribution characteristics within the controlled environments.

Third, this study is still in the static analysis stage of microbial distribution map construction. The assessment of control efficacy may be affected by environmental conditions during specific monitoring periods, such as seasonal baseline microbial load. Follow-up research can further integrate multi-dimensional environmental impact data to develop dynamic analysis models for microbial distribution maps. For example: integrating microbial, particle, temperature, humidity, personnel flow, and material transfer data to build predictive risk assessment models for microbial contamination; introducing external geospatial information of the plant area to evaluate the impact of peripheral environments on microbial communities in clean zones; and using long-term monitoring data to identify seasonal fluctuations and evolution trends of microbial communities, thereby achieving a transition from static to dynamic risk assessment.

### Conclusions

This study systematically analyzes the structural characteristics and spatial distribution patterns of microbial communities in pharmaceutical cleanroom environments. The results indicate that (i) microbial abundance and diversity exhibit a significant gradient decline with increasing cleanliness grades. The composition of microbial communities in different clean areas shows notable correlations, with dominant populations primarily consisting of *Staphylococcus* and *Micrococcus*. Microorganisms in cleanroom environments generally possess surface colonization capabilities and facultative anaerobic characteristics. (ii) Particularly noteworthy, this study innovatively employs GIS to achieve the visualization and spatial localization of microbial contamination distribution, providing technical support for the precise identification of contamination sources. (iii) Building on this, a microbial contamination risk assessment framework is constructed by integrating spatial visualization analysis and microbial risk analysis strategies. Its applicability in identifying potential contamination sources and optimizing control measures is validated through specific case studies. This study not only deepens the understanding of microbial ecology in pharmaceutical cleanrooms but also further clarifies the necessity of spatial visualization in microbial contamination control research, offering a scientific foundation for future studies in related fields.

## Data Availability

The original contributions presented in the study are included in the article; further inquiries can be directed to the corresponding authors.
